# Histological Comparison of Buccal and Lingual Mucosa Grafts for Urethroplasty: Do They Share Tissue Structures and Vascular Supply?

**DOI:** 10.3390/jcm11072064

**Published:** 2022-04-06

**Authors:** Felix Campos-Juanatey, Ainara Azueta Etxebarria, Paola Calleja Hermosa, Sara Marcos Gonzalez, Eneko Alonso Mediavilla, Miguel Angel Correas Gomez, Jose Antonio Portillo Martin, Jose Luis Gutierrrez Baños

**Affiliations:** 1 Urology Department, Marques de Valdecilla University Hospital, Institute of Research Valdecilla (IDIVAL), 39011 Santander, Spain; 2 Pathology Department, Marques de Valdecilla University Hospital, Institute of Research Valdecilla (IDIVAL), School of Medicine, University of Cantabria, 39011 Santander, Spain; ainara.azueta@scsalud.es; 3 Urology Department, Marques de Valdecilla University Hospital, 39008 Santander, Spain; paola.calleja@scsalud.es (P.C.H.); eneko.alonso@scsalud.es (E.A.M.); 4 Pathology Department, Marques de Valdecilla University Hospital, 39008 Santander, Spain; sara.marcos@scsalud.es; 5 Urology Department, Marques de Valdecilla University Hospital, Institute of Research Valdecilla (IDIVAL), School of Medicine, University of Cantabria, 39011 Santander, Spain; miguelangel.correas@scsalud.es (M.A.C.G.); josepm117@gmail.com (J.A.P.M.); joseluis.gutierrez@scsalud.es (J.L.G.B.)

**Keywords:** anterior urethral strictures, reconstructive surgical procedures, tissue grafting, oral mucosa, histology, urologic surgical procedures

## Abstract

Comparable outcomes were published using a buccal mucosa graft (BMG) from the cheek and a lingual mucosa graft (LMG) from the sublingual area, for urethral augmentation or substitution. To date, no histological comparison between both grafts has been conducted. We histologically assessed BMG and LMG harvested during urethral surgeries, aiming to compare graft properties and vascular support. We conducted a prospective single cohort study, including oral mucosa urethroplasty patients. During surgery, graft dimensions and donor sites were collected, and a 0.5 × 0.5 cm sample was obtained from the prepared graft. Formalin-fixed paraffin-embedded samples were sliced at 4 micrometres (µm) and hematoxylin-eosin stained. Using a telepathology tool, all slides were digitalized and measured from 10× to 40× magnification. In each graft, global and individual layers thicknesses were assessed, including vascular density and area. Descriptive and comparative (parametrical and non-parametrical) statistical analysis occurred. We collected 57 grafts during 33 urethroplasties, with 30 BMG and 22 LMG, finally, included. The mean age was 56.6 (SD 15.2) years, and the mean graft length was 5.8 (SD 1.7) cm and the width was 1.7 (SD 0.4) cm. The median graft thickness was 1598.9 (IQR 1200–2100) µm, the mean epithelium layer was 510.2 (SD 223.7) µm, the median submucosa was 654 (IQR 378–943) µm. the median muscular was 477.6 (IQR 286–772) µm, the median vascular area was 5% (IQR 5–10), and the median adipose tissue area was 5% (IQR 0–20). LMG were significantly longer and narrower than BMG. Total graft thickness was similar between LMG and BMG, but the epithelium and submucosa layers were significantly thinner in LMG. The muscular layer was significantly thicker in LMG. Vascular density and vascular areas were not significantly different between both types of grafts. LMG showed significantly less adipose tissue compared with BMG. Our findings show LMG and BMG for urethroplasty surgeries share the same thickness and blood supply, despite having significantly different graft sizes as well as mucosal and submucosal layers thickness.

## 1. Introduction

Urethral reconstruction still poses a challenge for urologists. A wide variety of techniques are described, when urethral substitution or augmentation is required. Aside from local flaps, many tissues have been proposed for use as grafts: preputial or penile skin, non-hair bearing extra-genital skin, bladder mucosa, colonic mucosa, or oral mucosa. Recently, some consensus was reached, and according to EAU and AUA Guidelines, oral mucosa should be the tissue of choice when urethral grafting is advised [1,2]. This recommendation is due to the good urethroplasty results related to oral mucosa properties: easy access for harvesting the graft, concealed donor site, constant availability, resistance to infection, and excellent tolerance to a wet environment.

A buccal mucosa graft (BMG) from the inner cheek was first proposed [3] and is still more commonly used. A lingual mucosa graft (LMG) is slowly gaining acceptance, providing comparable results [1,4]. The lower lip is a common resource for pediatric surgeons, but it is not currently recommended in adult patients, as it is related to more sensory alterations and a risk of lower lip inversion [5,6]. Histological comparative studies have been conducted using animal models to test adaptation of different grafts and their response to urine exposure [7,8]. Assessment of graft characteristics in humans has been less frequently conducted, despite being more relevant.

Several studies have been published describing urethroplasty results and complications from oral mucosa harvesting. However, no histological comparison between both grafts has been conducted to date. In the present study, we histologically assessed BMG and LMG harvested during urethral surgeries, aiming to compare graft tissue properties and vascular supply.

## 2. Materials and Methods

### 2.1. Study Protocol and Patient Selection

A prospective single cohort study was designed. The research protocol was reviewed and approved by local Ethical Committee, and all included patients signed the specific informed consent form. The main objective was to compare the histological characteristics of grafts from the ventrolateral aspect of the tongue with those from the inner cheek, when prepared for their use in augmentation or substitution urethroplasties. Secondary objectives were assessing our consistency on graft preparation—keeping a comparable thickness, with uniform histological characteristics in each type of donor site—and comparing residual muscle fibers after preparation, as well as vascular density in both grafts.

We invited patients older than 18 years old, diagnosed with urethral disease, who were candidates for an augmentation or substitution urethroplasty. Exclusion criteria were patients with exclusive anastomotic urethral repair or augmentation using only local flaps, and when tissues other than oral mucosa were used for the urethral repair. Grafts previously harvested from a donor site were also not considered for analysis.

All patients were preoperatively evaluated by history, physical examination, uroflowmetry, post-void residual urine measurement, retrograde urethrography, and/or urethroscopy. Patients with active oral disease were submitted to a specialized evaluation by an oral and maxillofacial surgeon for clearance, before inclusion in the study.

Patients are explained both BMG and LMG, including specific postoperative side effects. Final decision on graft harvesting remains at the discretion of the surgeon, depending on intraoperative findings—final length of stricture, aspect of urethral plate—and considering previous oral harvesting sites. As a standard criterion, BMG are selected for wide defects, usually with a total length of 6 cm or less. For longer defects (i.e., panurethral repairs), LMG are generally preferred. Urethroplasty procedures and graft harvesting were performed by three separate consultant urologic surgeons with more than five years of experience in urethral reconstruction.

### 2.2. Surgical Technique

#### 2.2.1. Buccal Mucosa Graft Harvesting

A silicone jaw opener is placed at the contralateral side to the donor cheek, and 5-0 stay sutures are placed at the lip border for exposure. The Stensen duct must be identified, marked, and carefully preserved. A distance of at least 1 cm from the lip is kept, to minimize mouth-opening problems. The required graft is marked with a surgical pen. Submucosal injection of local anesthetic with epinephrine is routinely performed, to facilitate graft dissection and decrease bleeding. After a cold knife incision on the previously marked lines, the graft harvest is completed using sharp scissors. Bipolar energy is used for hemostasis, and the donor site is closed using absorbable 5-0 interrupted sutures.

#### 2.2.2. Lingual Mucosa Graft Harvesting

A plastic lip opener is placed for better exposure, and the apex of the tongue is pulled outside the mouth using a ring forceps or Babcock forceps. Donor site limits are the fimbriated fold medially, the lateral line of the tongue dorsally, and palatoglossal fold proximally. Separate grafts are obtained if bilateral LMG are required, preserving the distal tip of the tongue along with tongue frenulum. The graft is measured and marked, and submucosal local anesthetic with epinephrine is injected. Lateral edges are incised with a scalpel, and graft harvesting is completed using sharp scissors. Donor site is checked for hemostasis and closed using an absorbable 5-0 running suture.

#### 2.2.3. Graft Preparation, Sample Procurement, and Histological Analysis

**Figure 1:**
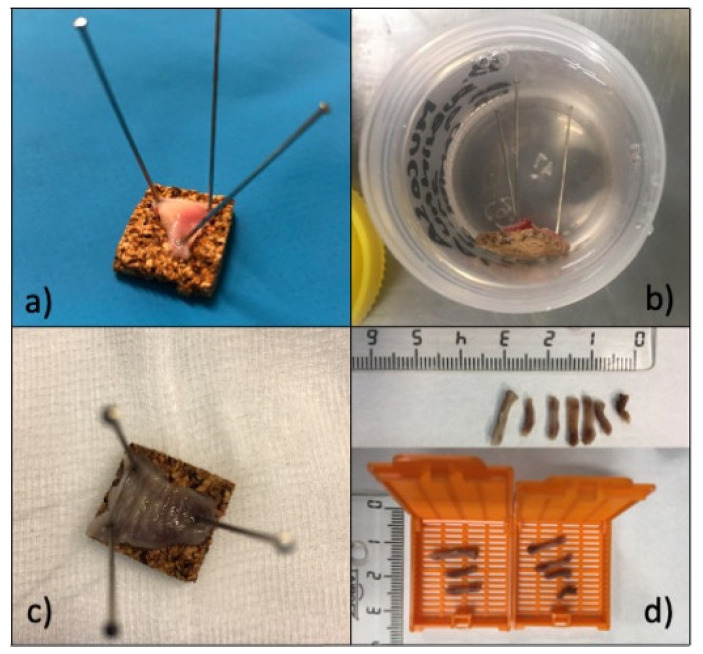
Graft sample preparation for histological analysis: (**a**) graft sample placed in a table and spread using pins; (**b**) sample in 10% buffered formalin; (**c**) final appearance after formalin fixation; (**d**) sample is sharply divided for paraffin embedding.

After harvesting, grafts are spread and prepared, removing muscular fibers and adipose tissue until they have a whitish appearance [9]. When ready, grafts should not be translucent, in order to preserve the submucosal layer. Before suturing the graft in the urethra, a representative tissue sample of 0.5 × 0.5 cm is obtained using a cold knife—Figure 1—and the graft sample is spread using pins and sent for histopathological analysis. Initially, tissue is placed in 10% buffered formalin, with a minimum fixing time of 6 h and a maximum of 24 h. The sample is then processed with alcoholic dehydration and paraffin embedding. Histological 4-µm thickness sections are obtained by microtomy and subsequently stained with hematoxylin-eosin.

**Figure 2:**
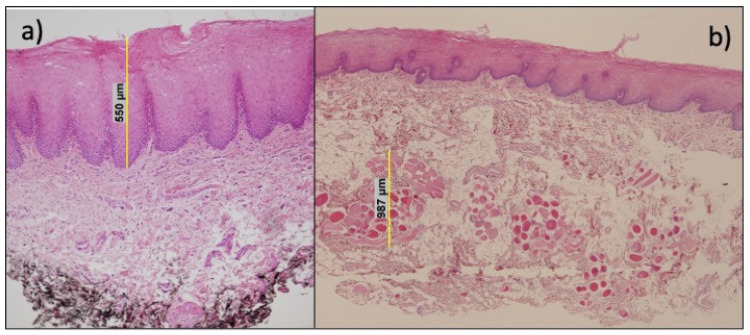
Histological preparation of oral mucosa: (**a**) Epithelium layer measurement; (**b**) muscle area thickness.

Using an optic microscope under 10× to 40× magnification, the issues were evaluated. Moreover, a telepathology accessory tool (Pannoramic 250 scanner. 3DHISTECH Ltd., Budapest, Hungary) was used for capture images. The following variables were measured using specific histology analysis software (ClinicalViewer. 3DHISTECH Ltd., Budapest, Hungary): total graft thickness (µm); epithelium (µm), submucosa (µm) and muscular (µm) layers thickness; vascular density (nº plexus/mm^2^), vascular area (% of the area of lamina propria occupied by vessels); and adipose tissue area (%)—Figure 2.

**Figure 3:**
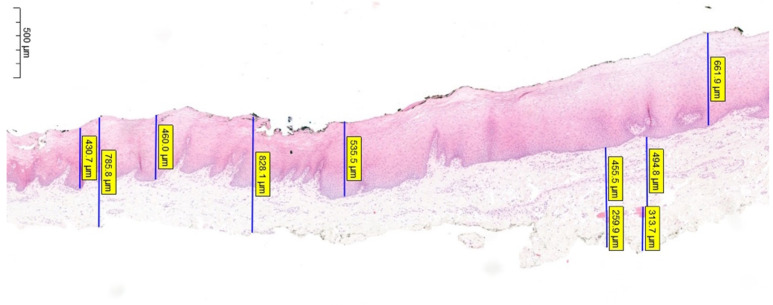
Histological preparation of oral mucosa (BMG) showing multiple measurements of different layers thicknesses.

In each sample, 3 independent measures of all parameters were performed by 2 separate dedicated uropathologists to minimize bias. We used the mean value of these 6 determinations as final measure for each parameter—Figure 3.

### 2.3. Clinical Information

#### Clinical Information, Data Storage and Statistical Analysis

Patient (age) and graft characteristics (donor site, graft length and width) were recorded for each graft during urethroplasty procedure, and recorded in the operation notes. Data were prospectively collected and securely stored in an Access database.

Variability of grafts characteristics was assessed using the Kolmogorov–Smirnoff test. A Student’s t-test for independent samples was used to compare normally distributed variables, while a Mann–Whitney (Wilcoxon Rank-Sum) test was used for non-parametric comparisons. Significant difference was considered when *p* < 0.05. Statistical analysis was performed using STATA 13.1 software for Mac (StataCorp, College Station, TX, USA).

## 3. Results

Between February 2019 and August 2021, a total of 57 grafts (23 LMG and 34 BMG) were harvested during 33 urethroplasties. Median patient age was 56 (IQR 44.1–66.8) years. A median of two (IQR 1–2, Range 1–4) grafts were obtained per procedure. During laboratory procedures, five samples (8.8%) were lost. In total, 30 BMG and 22 LMG were, finally, included in histological analysis.

Global characteristics of analyzed samples are described in Table 1.

Six samples (11.5%) did not contain any muscle fibers—five BMG and one LMG. Sixteen samples (30.8%) did not contain any adipose tissue. Minimal inflammatory infiltrate of the submucosal tissue was identified in 14 samples (24.6% of samples). Only two samples, both from the same patient, presented important inflammatory changes-undetected during urethroplasty procedure. This patient had an uneventful postoperative course, and the reconstructive procedure was successful throughout more than one year of follow-up. Human papillomavirus (HPV) was isolated in two cases by PCR techniques. No oral nor urethral macroscopic HPV-related lesions have been encountered in these patients to date.

The main histological differences between grafts are summarized in Table 1. LMG were significantly longer (2.2 cm 95%CI 1.5–2.9) and narrower (−0.4 cm, 95%CI −0.2–−0.5) than BMG. Total graft thickness was similar between both grafts. However, the epithelium layer was significantly thinner in the LMG group compared with BMG (−161.5 µm, 95%CI −40.9–−282.1). The subepithelial layer was also significantly thinner in LMG (−366.5 µm, 95%CI −114–−619). Conversely, the muscular layer was significantly thicker in the LMG group (298.8 µm, 95%CI 19.7–617.4). Vascular density and vascular areas were not significantly different between both types of grafts. LMG showed significantly less adipose tissue compared with BMG (−14%, 95%CI −3–−25.2).

## 4. Discussion

Oral mucosa is nowadays accepted as the tissue of choice for urethral augmentation or substitution [1]. Although Humby was credited with the first description of oral mucosa during an urethroplasty in 1941 [10], it was not until 1992, when Bürguer and Dessanti published their studies, which popularized oral tissues for urological reconstruction [3,11]. Following studies on BMG reported long-term urethral patency rates between 75.6% and 92% [12,13,14,15,16], even in panurethral strictures [17].

Although BMG are easily harvested, these second surgical sites could lead to short- and long-term complications—such as oral tightness and numbness—especially if longer or wider grafts are obtained. Aiming to reduce donor site morbidity, while exploiting another source of oral tissue, LMG was proposed [18] and has become increasingly common [19]. The ventrolateral area of the tongue has similar mucosa to the rest of the oral cavity, and this part of the tongue has no specific functional features [18], limiting the risks associated with graft harvesting. Theoretically, almost half of the lingual tissue could be obtained without causing functional limitations [20]. Simonato et al. described using LMG in urethral repair for the first time in 2006 [18], with a promising 79% success rate after a median 21-month follow-up, in a series of 29 LMG urethroplasties [21]. The mean length and width of LMG were 5.3 (3–9 cm) and 1.5 cm, respectively. After these initial studies, other authors reported success rates between 83–93%, proving the reproducibility, safety, and efficacy of LMG in urethral surgery [22,23,24,25,26]. LMG are particularly useful when long grafts are required, as 7–8 cm longitudinal pieces can be easily obtained from each ventrolateral aspect of the tongue [18,21,27]. In our study, LMG have a median length of 7 cm, being significantly longer than BMG.

Comparative studies of BMG and LMG urethroplasties showed similar outcomes in terms of urethral patency, with reported success rates of 76%–86% and 75%–89% for BMG and LMG urethroplasties, respectively [28,29,30,31]. However, regarding donor site complications, some differences appeared. A recent metanalysis on oral complications, including 632 patients from 12 comparative studies (4 RCTs and 8 non-randomized), reported a higher proportion of patients after LMG with tongue protrusion and speaking difficulties (RR 12.9 and 6.96, respectively). Conversely, BMG patients had more incidence of early postoperative swelling and mouth tightness [4]. These differences are probably related to their anatomical function, as the tongue plays an important role in speech, while the cheek is stretched during chewing and mouth opening [27]. Although most complications and donor site pain would resolve within first postoperative weeks, long-term oral sequelae (after three months) have been reported. Mouth tightness has been reported in up to 24% of patients six months after BMG obtention, as opposed to 3.4% in LMG [30]. However, in a retrospective study, LMG was associated with difficulty in tongue movements, numbness in donor site and speaking difficulties in 6.2%, 4.9%, and 2.5% of patients, respectively, even after 12 months following LMG urethroplasty [25]. In a recent study, it was reported that patients with a harvested LMG longer than 7 cm had a higher risk of oral morbidity compared to those with harvested LMG shorter than 7 cm (OR = 4.35) [32].

Several authors have investigated the characteristics and properties of oral mucosa, related to its suitability for urethral augmentation. Oral mucosa has a thick non-keratinized stratified squamous avascular epithelium and a well-vascularised lamina propria. Vessels infiltrate lamina propria through the submucosal layer, providing an effective mechanism for revascularization of the tissue when grafted. Histological architecture shows some similarities with urethral mucosa (stratified squamous epithelium) [33]. Moreover, oral mucosa has the ability to stretch and compress, using the papillae of connective tissue in the lamina propria that increase contact area between both layers. Additionally, oral mucosa is obviously adapted to a wet environment. Another important feature is a high resistance to infection, despite been continuously exposed to polymicrobial oral flora. This intrinsic resistance is secondary to antimicrobial peptide production from the epithelium, continuous epithelial cell exfoliation, which hinders colonization, and the mucosa-associated lymphoid tissue (MALT). For this reason, inflammatory infiltrates are rarely seen in a histological examination of the oral mucosa.

When compared with other tissue sources for urethral reconstruction, such as penile skin and bladder mucosa, oral mucosa has a much thicker epithelium with thinner lamina propria [34]. This would lead to cause sacculation and contracture at a lower rate in the postoperative period—if graft contracture is excessive, re-stricture will appear [35]. A similar interpretation may be considered in our comparison of BMG and LMG, that is, greater epithelial thickness may be an advantage for urethroplasty using a BMG. There is also an increased intrinsic vascularity of inner cheek buccal mucosa in comparison to other grafts. This increased vascularity is particularly dense immediately underlying the epithelial layer. Studies calculating the number of blood vessels in the dermis of skin and the buccal mucosa showed that oral tissue has a two-to-four-fold greater density of blood vessels. It is believed that a thin lamina propria would facilitate initial imbibition of a graft, and a highly vascularised donor tissue would promote inosculation and revascularization of transferred tissue [35].

Mokhless et al. [36] conducted a histological assessment of BMG used for staged urethroplasties. BMG samples were taken before grafting at the first stage and before the second stage. All BMG had completely taken within five days, and they showed minimal contraction. Analyzed grafts showed excellent vascularization, presenting minimal reactive changes in form of subtle acanthosis, epithelial hyperplasia, and keratosis, with lamina propria papillae elongation. These findings confirmed the extraordinary resistance of oral tissues to urine exposure and chronic irritation. Soave et al. [37] prospectively evaluated 22 patients with prior BMG urethroplasty. During re-do urethral repairs, collected samples were from the buccal mucosa area, native urethra healthy mucosa, newly harvested BMG, and fibrotic tissue from stricture area. Integrated BMG completely preserved its original architecture, maintaining a non-keratinised squamous epithelium, easily distinguished from urethral adjacent pseudostratified thin urothelium. They did not find differences in vascularization compared with newly obtained BMG. This finding contrasts with previous animal studies, showing extensive neovascularization in integrated buccal mucosa compared with newly harvested BMG [38].

To date, only one study assessed LMG histology. Song et al. performed a sublingual graft urethroplasty in 10 dogs [39]. After three months, tongue tissue was perfectly included in urethral mucosa, however, it was clearly distinguishable from original surrounding urethral tissues. The grafted area showed keratinized squamous epithelium with abundant neovascularization. They reported a 9.5% contraction rate, similar to previous reports in BMG studies.

For a taken graft, not only the native properties of transferred tissue are important, but also how the graft is prepared before fixation to the urethral bed. The sole study to date addressing oral grafts preparation for urethroplasty was published by Cavalcanti et al. [9]. They evaluated histological characteristics of oral mucosa prepared for urethroplasty in three different ways, assessing optimal grade of graft dissection for optimizing graft success. With an increasing grade of dissection, graft global thickness was gradually reduced by thinning the subepithelial layer, but without changing epithelium height. As the epithelium layer is avascular, depending on densely vascularised lamina propria, an aggressive dissection of graft could damage submucosal vascular plexus and, therefore, impair future graft survival. An intermediate dissection—leaving the graft with a whitish appearance—seems to offer the best outcomes due to the removal of adipose tissue while preserving the subepithelial layer [9].

In our study, the previously described whitish appearance was an aim during graft preparation before transferring. However, in comparison, our BMG group showed a similar epithelium layer, but a slightly thinner subepithelial layer and overall thickness, probably in relation to a greater degree of graft trimming (Table 2).

The fact that BMG contains more vascular tissue—without significant differences—may represent an advantage for graft survival, but on the other hand, a thinner submucosal layer of LMG may present an advantage for graft survival [35].

Other factors than preparation have been reported to influence graft thickness. According to Vandana et al. [40], epithelium thickness is directly related to male gender and indirectly associated with age. Recently, Kurtzman et al. [41] evaluated BMG histology, correlating it with preoperative oral health in a series of 51 patients. They evidenced that, as oral health worsened (scored by different questionnaires), average epithelial and lamina propria thickness are decreasing, as well as the delta stretch of the graft. Such findings raise the question about graft quality in patients with compromised oral health as well as possible relevance with urethroplasty outcomes. However, further studies are needed to clarify such a question.

Before recommending oral grafting, a thorough head and neck examination is advised, to avoid transferring diseased tissues to the recipient site. In patients with specific clinical conditions, such as leukaemia, mucositis associated with head and neck cancer therapy, oral lichen planus, pemphigus vulgaris, recurrent aphthous stomatitis, and leukoedema, the oral graft harvest is strictly contraindicated [33]. Ethanol abuse increases oral mucosa permeability, and may induce dysplastic changes in oral mucosa, contraindicating graft harvest. Heavy smokers also warrant careful examination of oral mucosa for dysplastic changes, at it is heavily associated with malignancy. Moreover, in patients who regularly use NSAIDS, ACE inhibitors, or angiotensin receptor blockers, oral grafts are relatively contraindicated, as they have been associated with angioedema of oral mucosa. Other medications—including clindamycin, ibuprofen, barbiturates, and captopril, among others—can cause erythema multiforme or lichenoid lesions. Both of these conditions contraindicate oral harvest [33].

Recently, the importance of oral cavity evaluation, particularly if risk factors for dysplasia or malignancy are present, was highlighted by Massimo et al. [42], publishing a case report of two patients presenting squamous cell carcinoma in BMG after urethroplasty. In none of them an oral malignancy was demonstrated, so it was considered that malignancy appeared in BMG after urethroplasty. Both patients featured risk factors for oral—and urethral—malignancies: smokers, chronic HPV infection, and chronic inflammation after urethroplasty. In our series, HPV was isolated in two grafts. However, to date, no oral or urethral lesions have been demonstrated in such patients.

Our study presents some limitations that should be acknowledged. Final grafts number was affected by initial handling problems, with up to 9% of samples being unsuitable for histological assessment. Choice and size of LMG and BMG are affected by surgeon preference in each urethral case, which could influence the results of our series. As strengths, we should mention the double uropathologist measurement of histological characteristics in each sample, minimizing bias. Finally, we are presenting pure histological analysis of LMG for urethral grafting—to the best of our knowledge, for the first time in the literature—but our study lacks clinical correlation. All of our patients are being prospectively followed, and we expect to compare urethroplasty outcomes, relating them with graft type and characteristic in due time.

## 5. Conclusions

LMG are usually longer and narrower than BMG when harvested during urethral surgeries. Both oral grafts share global thickness and vascular density and area. However, LMG present thinner epithelium and submucosa, and a lower percentage of adipose tissue compared to BMG. Conversely, LMG show thicker muscular layer than BMG. Further studies with long-term follow-up are required to understand if histological differences are relevant for urethroplasty outcomes.

## Figures and Tables

**Table 1:** Global characteristics of samples and histological differences between BMG and LMG.

Variables, Units	Global	BMG	LMG	*p*
Age, years (median, IQR)	56 (44.1–66.8)	57.7 (50.7–74.9)	54.9 (41.5–66.7)	0.320
Length, cm (median, IQR)	5.5 (4.5–7)	5 (4.5–5.5)	7 (6.5–8)	0.000
Width, cm (median, IQR)	1.5 (1.5–2)	2 (1.5–2)	1.5 (1.5–1.5)	0.000
Total thickness, µm (median, IQR)	1598.9 (1200–2100)	1692.8 (1382.4–2194.8)	1347 (1005.7–1650)	0.1
Epithelium thickness, µm (mean, SD)	510.2 (223.7)	576.8 (234.3)	415.2 (171.5)	0.009
Submucosal thickness, µm (median, IQR)	654 (378.6–943.3)	823.6 (470.4–1026.2)	438 (267.6–654)	0.005
Muscular thickness, µm (median, IQR)	477.6 (286.8–772.5)	324.5 (205.5–483)	572.5 (483.2–878)	0.003
Vascular area, % (median, IQR)	5 (5–10)	5 (5–10)	5 (2–10)	0.231
Vascular density, nº plexus/mm^2^ (median, IQR)	5 (2–7)	4 (3–7)	3 (2–6)	0.297
Adipose tissue, % (median, IQR)	5 (0–20)	10 (2–40)	2 (0–10)	0.018

**Table 2:** Comparation of the histological measurement of graft layers between Cavalcanti et al. [9] and the current series.

	Cavalcanti-Group 2-	Present Series-BMG-
Graft thickness, µm	Mean 1830 (SD 520)	Median 1692.8 (IQR 1382.4–2194.8)
Epithelium, µm (mean)	530 (SD 160)	576.8 (SD 234.3)
Subepithelial layer, µm	Mean 1220 (SD 580)	Median 823.6 (IQR 470.4–1026.2)

## Data Availability

Not applicable.
